# High Conservatism in the Composition of Scent Gland Secretions in Cyphophthalmid Harvestmen: Evidence from Pettalidae

**DOI:** 10.1007/s10886-012-0108-8

**Published:** 2012-04-04

**Authors:** Günther Raspotnig, Julia Schwab, Ivo Karaman

**Affiliations:** 1 Institute of Zoology, Karl-Franzens University, Universitätsplatz 2, 8010 Graz, Austria; 2 Research Unit of Osteology and Analytical Mass Spectrometry, Medical University, University Children’s Hospital, Auenbruggerplatz 30, 8036 Graz, Austria; 3 Department of Biology and Ecology, Faculty of Science, University of Novi Sad, Trg Dositeja Obradovica 2, 2100 Novi Sad, Serbia

**Keywords:** Cyphophthalmi, Chemical defense, Methylketones, Naphthoquinones, Arachnida, Opiliones

## Abstract

The scent gland secretion of *Austropurcellia forsteri* was analyzed by gas chromatography–mass spectrometry, providing the first description of the secretion chemistry in the cyphophthalmid family Pettalidae. The secretion contained a total of 21 compounds: About 60% of the whole secretion consisted of a series of saturated, mono-unsaturated and doubly unsaturated methylketones, from C_11_ to C_15_, with a cluster of saturated and mono-unsaturated C_13_-methylketones dominating. A second fraction included several naphthoquinones such as 1,4-naphthoquinone (ca. 20% of secretion), 6-methyl-1,4-naphthoquinone (ca. 17%), and minor amounts of chloronaphthoquinones (ca. 2%). When compared with scent gland compositions of other representatives of cyphophthalmids (e.g. from families Sironidae and Stylocellidae), a highly conservative chemistry of cyphophthalmid secretions is apparent, based on a restricted number of methylketones and naphthoquinones.

## Introduction

The Cyphophthalmi represent the smallest suborder of harvestmen, currently comprising about 190 described species in six families (Giribet et al., 2012). Like all harvestmen, the Cyphophthalmi possess prosomal defensive (“scent”) glands, which are strikingly developed across this suborder. When a leg is grasped by forceps, many (if not all) cyphophthalmid species expel droplets of scent gland secretion from dorso-laterally protruding ozophores, dab each droplet with the tip of one leg (mostly leg II), and transfer the secretion to the forceps. This behavior has been described as “leg dabbing,” and it clearly identifies cyphophthalmid scent gland secretions as defensive (Juberthie, 1961). The chemistry of cyphophthalmid secretions, however, has been poorly studied. Raspotnig et al. (2005) reported on secretions of two species of the family Sironidae, *Cyphophthalmus duricorius* (from Europe) and *Siro exilis* (from USA), and Jones et al. (2009) studied the chemistry of an undescribed Sulawesian stylocellid. All three species produce multicomponent secretions that, although distinguishable, are based on common chemistry of acyclic methylketones and naphthoquinones. No chemical analyses on the secretions of other cyphophthamid species have been performed.

Here, we investigated the chemistry of the scent gland secretion of a representative of a further cyphophthalmid family, the Pettalidae.

## Methods and Materials

### Species Collection

Twelve adults (5 males, 7 females) and 6 juvenile specimens of *Austropurcellia forsteri* (Juberthie, 2000) (Pettalidae) were collected from leaf litter at Cape Tribulation (Queensland, Australia). This species, originally included within the genus *Neopurcellia* by Juberthie (2000), has been transferred to the genus *Austropurcellia* on the basis of molecular data (Boyer and Giribet, 2007), and is considered to represent a member of a monophyletic clade of pettalids in the northeast of Australia. Species determination was performed by the original description of Juberthie (2000).

### Extraction and Analysis of Secretions

Scent gland secretions were obtained either by dabbing secretion from ozopores on filter paper or by whole body extraction of single individuals. Extracts were analyzed by gas chromatography–mass spectrometry (GC-MS), using a Trace GC2000 (with a ZB-5 capillary column, 30 m x 0.25 mm i.d., x 0.25 μm film thickness and helium at 1.2 ml.min^-1^) coupled to a DSQ MS (ion source at 200°C; transfer line at 310°C). The temperature of the GC oven was programmed from 50°C (1 min. delay) to 300°C at 10°C.min^-1^, then 5 min at 300°C. Areas of individual peaks were integrated and expressed as % peak area of a whole extract.

### Reference Compounds

Compounds were identified by comparison of mass spectral and gas chromatographic data to synthetic standards and by comparison to already identified components in *Cyphophthalmus* extracts (Raspotnig et al., 2005). Synthetic 1,4-naphthoquinone, undecan-2-one, dodecan-2-one, and tridecan-2-one were purchased from Sigma (Vienna, Austria), and 4-chloro-1,4-naphthoquinone was synthesized as described in Raspotnig et al. (2005). As a reference source for 6-methyl-1,4-naphthoquinone, we used the scent gland secretion of *Phalangium opilio* (Wiemer et al., 1978).

## Results

### Chemical Analysis of Scent Gland Secretion

Chromatograms of secretion-loaded filter papers or whole body-extracts of individuals showed identical chromatographic profiles. Therefore, further chromatograms were obtained using whole body-extracts of individuals.

**Fig. 1:**
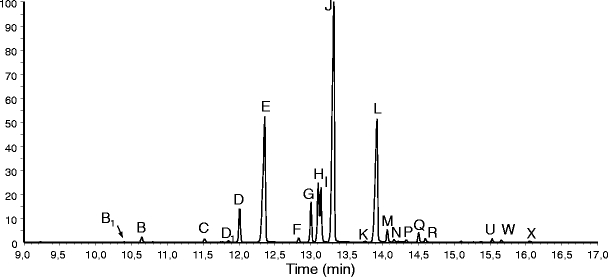
Total ion chromatogram (y-axis shows relative peak heights) from scent gland secretions of *Austropurcellia forsteri*. Peak identities are listed in Table 1. The profile was obtained by dabbing the secretion from the ozopores of an individual female on filter paper. Note that peaks A, O, S, T, and V (see Raspotnig et al., 2005) are missing in the secretion of *A. forsteri*, but may be present in other cyphophthalmids

In total, 21 compounds were detected (Table 1; Fig. 1), all of which were either methylketones (16 compounds B_1_, B, C, D_1_, D, F, G. H. I, J, K, M, N, P, Q, U, W) or naphthoquinones (four compounds: E, L, R, and X). Nineteen of the compounds had previously been identified in *Cyphophthalmus duricorius* and *Siro exilis* (Raspotnig et al., 2005). The two remaining compounds (B_1_ and D_1_) had not been detected in our previous study, but appeared to be mono-unsaturated homologs of undecan-2-one (compound B) and dodecan-2-one (compound C), and thus were tentatively identified as undecen-2-one (B_1_) and dodecen-2-one (D_1_), respectively. These identifications were supported by molecular ions two mass units lower (M^+^ at *m/z* 168 and 182, respectively) than those of the saturated compounds, along with a strong *m/z* 58 (McLafferty-rearrangement, indicating a methylketone).

**Table 1:** Scent gland secretion profiles of *Austropurcellia forsteri*

Peak*	RI**	Identified as	Relative abundance (% of whole secretion ± SD)		
			males (*N* = 5)	females (*N* = 7)	juvenile (*N* = 6)
B_1_	1276	undecenone	0.15 ± 0.13	0.23 ± 0.24	0.02 ± 0.03
B	1293	undecan-2-one	1.34 ± 0.42	1.12 ± 0.58	0.55 ± 0.23
C	1358	dodecan-2-one (branched isomer)	0.43 ± 0.03	0.34 ± 0.16	0.25 ± 0.08
D_1_	1383	dodecenone	0.14 ± 0.12	0.37 ± 0.27	0.22 ± 0.18
**D**	1395	**dodecan-2-one**	**3.58 ± 1.88**	**5.80 ± 2.34**	**6.11 ± 2.77**
**E**	1421	**1,4-naphthoquinone**	**20.84 ± 1.86**	**19.33 ± 2.81**	**19.47 ± 3.48**
F	1459	tridecan-2-one (branched isomer)	0.46 ± 0.09	0.51 ± 0.17	0.66 ± 0.13
**G**	1473	**6-tridecen-2-one**	**3.18 ± 0.32**	**3.41 ± 0.80**	**2.41 ± 0.82**
**H**	1481	**7-tridecen-2-one**	**6.90 ± 1.55**	**6.11 ± 1.20**	**9.83 ± 3.06**
**I**	1484	**tridecadienone**	**3.70 ± 1.19**	**6.26 ± 2.20**	**4.39 ± 1.39**
**J**	1498	**tridecan-2-one**	**37.98 ± 1.21**	**34.51 ± 1.58**	**38.55 ± 6.87**
K	1534	tetradecanone (branched isomer 1)	0.16 ± 0.04	0.09 ± 0.05	0.04 ± 0.05
**L**	1547	**6-methyl-1,4-naphthoquinone**	**17.32 ± 1.37**	**16.90 ± 1.68**	**13.13 ± 1.55**
M	1560	tetradecanone (branched isomer 2)	1.29 ± 0.13	1.67 ± 0.45	1.17 ± 0.28
N	1568	tetradecanone (branched isomer 3)	0.25 ± 0.04	0.24 ± 0.08	0.26 ± 0.10
P	1583	tetradecenone	0.16 ± 0.03	0.18 ± 0.06	0.11 ± 0.09
Q	1597	tetradecan-2-one	0.97 ± 0.23	0.95 ± 0.22	0.77 ± 0.17
R	1604	4-chloro-1,2-naphthoquinone	0.46 ± 0.50	0.97 ± 0.59	1.50 ± 1.19
U	1687	pentadecenone	0.21 ± 0.09	0.40 ± 0.19	0.21 ± 0.12
W	1699	pentadecan-2-one	0.24 ± 0.05	0.21 ± 0.05	0.25 ± 0.05
X	1736	6-methyl-4-chloro-1,2-naphthoquinone	0.24 ± 0.30	0.92 ± 1.09	0.12 ± 0.19

*Peak annotation according to Raspotnig et al. (2005). For completeness, the “missing” compounds in this list (present in *Cyphophthalmus* and *Siro*, but not in *Austropurcellia*) are: A (acetophenone), O (isomeric tetradecenone), S (pentadecadienone), T (pentadecatrienone), and V (unknown). **Retention index, calculated as ${\hbox{R}}{{\hbox{I}}_{\rm{x}}} = {1}00{{\hbox{n}}_0} + ({1}00{{\hbox{t}}_{\rm{x}}}--{1}00{\hbox{t}}{{\hbox{n}}_0})/({\hbox{t}}{{\hbox{n}}_1} - {\hbox{t}}{{\hbox{n}}_0})$, with x = target compound; t_x_ = retention time of target compound; n_0_ = number of carbons in the alkane eluting directly before x; tn_0_ = retention time of alkane directly eluting before x; tn_1_ = retention time of alkane eluting directly after x. All tetradecanones (K, M, N, and Q; three of these probably methyl-branched) as well as the unsaturated ketones (B_1_, D_1_, P, and U) are most likely also 2-ketones, as indicated by *m/z* 58. Main compounds (> 3% of whole secretion) in bold

Chemical profiles of individuals showed only minor intraspecific variation (Table 1). Profiles of males and females were not separable by a principal component analysis (data not shown). Profiles of juveniles did not show major differences from adults, although early instars, in particular, frequently lacked trace components found in adult secretions. Profiles of late instars were indistinguishable from those of adults.

## Discussion

### Is There a Common Cyphophthalmid Scent Gland Chemistry?

Along with the present report, data on the scent gland chemistry of four cyphophthalmid species across three families are now available. The secretions of all four species show very similar composition, based exclusively on methylketones and naphthoquinones, largely comprising even the same compounds. Since these data are derived from different and possibly distantly related families (e.g., Giribet et al., 2012), this suggests a homogeneous chemistry of the scent glands of cyphophthalmids.

Methylketones, especially those ranging from C_11_-C_15_, were abundant in all secretions, with tridecan-2-one being the main component in *A. forsteri* (35%) and in the Sulawesian stylocellid analyzed by Jones et al. (2009; 50%), as well as in the two sironids (Raspotnig et al., 2005; 20%). Since methylketones have not been detected in any non-cyphophthalmid opilionid, they may constitute an autapomorphy of cyphophthalmid scent glands. In contrast, naphthoquinones are well-known from the scent gland secretions of both Cyphophthalmi and Palpatores: 1,4-Naphthoquinone and 6-methyl-1,4-naphthoquinone have been reported from the scent glands of phalangiid Eupnoi (Wiemer et al., 1978) and from Dyspnoi (Raspotnig et al., 2010). Although their occurrence in the latter groups is based on single investigations, we know that these components show a wide distribution among the Palpatores (Raspotnig, unpublished). In these terms, naphthoquinones may be regarded as synapomorphic for Cyphophthalmi and Palpatores, possibly indicating a common ancestry of these two suborders.

Interestingly, and as in sironids, *A. forsteri* also produces two highly unusual chloronaphthoquinones in its scent gland secretion, although in low abundance (each less than 1% of secretion). These chloronaphthoquinones were detected previously in both *Cyphophthalmus* and *Siro* (Raspotnig et al., 2005), but were lacking in the Sulawesian stylocellid. Jones et al. (2009) considered these compounds a possible autapomorphy of sironids, but their occurrence in *Austropurcellia*, although in low amounts, contradicts this idea.

### Are Cyphophthalmid Secretions Ancient?

According to Boyer and Giribet (2007) and Giribet et al. (2012), pettalids form a monophyletic clade, and their extant diversity is considered the result of diversification processes that started over 180 million years ago, paralleling the break-up of Gondwana into the present continents and landmasses. The origin of pettalids, however, may be more ancient and may date back to the Carboniferous era. Some authors regard pettalids as basally branching cyphophthalmids, possibly representing the sister group to all other cyphopthalmid families (Giribet et al., 2012). If this is correct, methylketones and naphthoquinones may have already been present in a common ancestor of cyphophthalmids, with this ancestral chemistry being largely unchanged over 300 million years. This presumed chemical conservatism of cyphophthalmid secretions is intriguing, and implies high survival benefits associated with these secretions that, essentially, could have contributed to the long-term evolutionary success of the group.
